# Glycoprotein M6A Suppresses Lung Adenocarcinoma Progression via Inhibition of the PI3K/AKT Pathway

**DOI:** 10.1155/2022/4601501

**Published:** 2022-11-11

**Authors:** Qian Zhang, Shuangshuang Deng, Qinchuan Li, Guangxue Wang, Zhongliang Guo, Dongyi Zhu

**Affiliations:** ^1^Department of Respiratory Medicine, East Hospital, Tongji University School of Medicine, Shanghai 200123, China; ^2^Department of Respiratory Medicine, Shanghai East Clinical Medical College, Nanjing Medical University, Shanghai 200123, China; ^3^Department of Pathology, East Hospital, Tongji University School of Medicine, Shanghai 200123, China; ^4^Department of Cardiothoracic Surgery, East Hospital, Tongji University School of Medicine, Shanghai 200120, China; ^5^Research Center for Translational Medicine, East Hospital, Tongji University School of Medicine, Shanghai 200120, China

## Abstract

Lung adenocarcinoma is the most common subtype of lung cancer and has high morbidity and mortality. Glycoprotein M6A (GPM6A) is a neuronal membrane glycoprotein reported to be related with cancer. However, studies on GPM6A in lung adenocarcinoma are rare. This study aimed to investigate the role of GPM6A in lung adenocarcinoma and its potential mechanism. GPM6A mRNA expression was analysed in 33 types of cancers using The *Cancer* Genome Atlas (TCGA) datasets. It was compared among normal lung tissues, lung adenocarcinoma tissues, and adjacent tissues using the Oncomine database. Real-time quantitative polymerase chain reaction (RT-qPCR) was performed to detect GPM6A expression in human lung adenocarcinoma cell lines (A549 and H1299) and normal pulmonary epithelial cells (BEAS-2B). When GPM6A was inhibited, cell proliferative capacity was detected by Cell Counting Kit 8 (CCK8), EdU, and colony formation assays. Cell migration ability was detected by wound healing and transwell assays. The expression of epithelial-mesenchymal transition (EMT) markers was detected by Western blotting (WB) and RT-qPCR. When GPM6A was overexpressed, cell proliferation and migration were detected again. Ten nude mice were subcutaneously injected with cells overexpressing GPM6A or empty vector, and the tumor size was recorded on day 14 and then measured every 3 days thereafter. The final tumor weight was measured on day 36. Furthermore, the expressions of phosphoinositide 3-kinase (PI3K), phosphorylated PI3K, AKT, and phosphorylated AKT were detected by WB. Results showed that GPM6A mRNA expression decreased in 15 types of tumors in TCGA dataset. GPM6A expression was lower in lung adenocarcinoma than in normal lung tissues or adjacent tissues in the Oncomine dataset. Similar results were found in lung adenocarcinoma cells. The function study showed that GPM6A downregulation enhanced the proliferation, migration, and EMT of lung adenocarcinoma cells, while GPM6A upregulation inhibited their development. The xenograft results suggested that GPM6A upregulation delayed tumor growth and reduced tumor weight. Moreover, WB showed that GPM6A knockdown activated the PI3K/AKT pathway, while GPM6A upregulation inhibited the activation of the PI3K/AKT pathway. In conclusion, GPM6A suppresses lung adenocarcinoma progression via inhibition of the PI3K/AKT pathway. Thus, GPM6A could be a possible treatment target for lung cancer therapy.

## 1. Introduction

Lung cancer is a common cancer that is the leading cause of death globally, with approximately 2.2 million new cases and 1.8 million deaths reported in 2020 [1]. Further, it is the second most commonly diagnosed malignancy (11.4% of total cancer cases) and the leading cause of cancer death (18% of total cancer-related deaths) [1]. Lung adenocarcinoma is a common subtype of lung cancer. It causes first and second-order cancer morbidity to men and women in China [2]. Advanced lung adenocarcinoma is primarily treated with targeted therapies for specific gene mutations; however, drug resistance is an inevitable issue [3, 4]. Checkpoint immunotherapy has been approved as first-line therapy for patients with Non-small cell lung cancer (NSCLC) expressing programmed cell death-ligand 1 (PD-L1) (tumor proportion score (TPS) ≥ 1%) [5, 6]. However, it can also cause a spectrum of immune-related adverse events including colitis, hypophysitis, pneumonitis, thyroiditis, and inflammatory arthritis [7].

M6 is a neuronal membrane glycoprotein that belongs to the proteolipid protein gene family. It is divided into two subtypes: glycoprotein M6A (GPM6A) and M6B (GPM6B). It was first found in the developing murine embryonic central nervous system (CNS), and it mainly expressed in both neonatal and adult CNS regions [8], participating in the pathological process of CNS. Fuchsova et al. found low GPM6A expression in the hippocampus of depressed suicide victims [9]. Ma et al. reported that GPM6A showed a trend of dysregulation in schizophrenia [10]. Gregor et al. also revealed that high expression of GPM6A was associated with learning disability and behavioral anomalies [11]. Recent studies have shown the association of GPM6A with cancer. However, the function of GPM6A in cancers is controversial. Charfi et al. indicated that GPM6A was highly expressed in lymphoid leukemia and promoted the transformation and proliferation of fibroblast cell (NIH/3T3), which may act as a candidate biomarker for human B-cell malignancies [12]. Ye et al. also revealed that GPM6A expression in the protein level was higher in undifferentiated or minimally differentiated colorectal carcinoma tissues than in highly differentiated colorectal carcinoma tissues, which suggested that GPM6A was related to poor outcomes in colorectal cancer [13]. However, Liu et al. showed that GPM6A expression was lower in hepatocellular carcinoma (HCC) than para-carcinoma tissues. They reported that circCCNB1 silencing which acted as a miR-106b-5p sponge inhibited GPM6A expression to promote HCC progression and activation of the AKT/ERK signaling pathway *in vivo* and *in vitro* [14]. Jiang et al. also showed that miR‐22 overexpression could inhibit the migration of small cell lung cancer cells, and elevated GPM6A was observed in cells with miR‐22 overexpression [15]. Chen et al. analysed the gene expression of 100 normal specimens and 94 lung cancer samples from the Gene Expression Omnibus database, and GPM6A was identified as a differentially expressed gene. GPM6A expression was significantly lower in lung cancer samples than in normal specimens [16]. These results suggested that GPM6A was suppressed in cancers. However, evidence on the role of GPM6A in lung adenocarcinoma is limited.

In this study, we analysed GPM6A in 33 types of tumors in TCGA dataset and also the GPM6A expression between lung adenocarcinoma and adjacent tissues in the Oncomine database. Then, we explored the function of GPM6A in the development of lung adenocarcinoma *in vitro* and *in vivo* and the potential mechanism of GPM6A in lung adenocarcinoma cells. Importantly, we demonstrated that GPM6A suppressed lung adenocarcinoma progression via inhibition of the PI3K/AKT pathway.

## 2. Methods

### 2.1. Biological Information Data

GPM6A gene expression was compared between tumor and corresponding nontumor tissues in 33 types of cancers from The *Cancer* Genome Atlas (TCGA) database using the GEPIA web tool (http://gepia.cancer-pku.cn). Hou Lung and Su Lung data were downloaded from the Oncomine database (https://www.oncomine.org) (the website was taken offline 17 January 2022) to analyse GPM6A expression. Hou Lung data were used to compare GPM6A expression between normal lung and lung adenocarcinoma tissues, while Su Lung data were used to compare between lung adenocarcinoma and adjacent normal tissues. A survival analysis was performed between lung adenocarcinoma patients with high and with low GPM6A expression using the Kaplan–Meier plotter (http://kmplot.com/analysis/) (ID: 209469).

### 2.2. Cell Culture

Human lung adenocarcinoma cell lines (A549, H1299) and normal pulmonary epithelial cells (BEAS-2B) were acquired from the Chinese Academy of Science (Shanghai, China) cell bank. The cells were maintained in Dulbecco's modified Eagle's medium (DMEM; Hyclone, Camarillo, CA, USA) supplemented with 10% fetal bovine serum (FBS; Gibco, Grand Island, NY, USA) at 37°C in a 5% CO_2_ incubator [17]. The DMEM was replaced two to three times per week.

### 2.3. RNA Isolation and Real-Time Quantitative Polymerase Chain Reaction

Total RNA (1 *μ*g) was extracted from the two cell lines using an EZ-press RNA purification kit (Epizyme Biomedical Technology, Shanghai, China). After mRNA was reverse transcribed to cDNA, we performed real-time quantitative polymerase chain reaction (RT-qPCR) using the SYBR Green mix (Thermo, Waltham, MA, USA). The 2^–ΔΔCT^ method was used to analyse gene expression. The primer sequences were obtained from Sangon (Shanghai, China) and listed in Table 1 [17].

### 2.4. Transfection

Small interfering RNAs (si-NC, si-GPM6A-1, and -2) were purchased from GenePharma (Shanghai, China). The siRNA sequences are listed in Table 1. The two cell lines were seeded in 6-well plates at 70% confluence and transfected with siRNA using Lipofectamine 3000™ (Thermo, Waltham, MA, USA). The transfection efficiency of siRNAs was detected after 48 h of transfection [17]. The overexpression plasmid packaged into lentivirus (HBLV-h-GPM6A-3xflag-ZsGreen-PURO) (Lv-GPM6A), and also, the control plasmids (HBLV-ZsGreen-PURO) (vector) were synthesized by Hanheng Biotechnology (Shanghai) Co., Ltd. The lentivirus was added to the cells after they reached 50% confluence in the 6-well plates. Puromycin (3-4ug/ml) was added to the two cell lines several times until the cells successfully transfected with the lentivirus.

### 2.5. Cell Counting Kit 8 Assay

For transient transfection, the cells were transfected with si-NC or si-GPM6A-1, -2 for 24 h and were inoculated in a 96-well plate at 3,000 cells per well. For constant transfection, the cells containing lentivirus were directly seeded in a 96-well plate. Cell Counting Kit-8 (CCK8) was used to detect cell proliferation at 0, 24, 48, and 72 h. The optical density value was detected at 450 nm absorbance.

### 2.6. EdU Assay

The cells were seeded in a 48-well plate for 24 h and were incubated with diluted EdU for 2 h. Then, 4% polyoxymethylene was used to fix cells for 30 min, and 0.5% TritonX-100 was added to permeate cells for 10 min. Finally, the cells were stained with Apollo and Hoechst. Images of the stained cells were taken under a fluorescence microscope. The ratio of EdU-positive cells was calculated as EdU expressed cells/DAPI stained cells in the same field×100%.

### 2.7. Colony Formation Assay

After transfection, the cells (500 per well) were seeded in 35-mm plates and cultured for 10 days. Giemsa was used to stain the cloned cells. More than 50 cells were clustered as a clone. The rate of colony formation was calculated as the number of cloned cells number/500 × 100%.

### 2.8. Wound Healing Assay

When the two cell lines reached 95% confluence in the 6-well plates, a 200-*μ*L tip was used to scratch a line. The cells were imaged at 0 and 24 h after the wound gap was formed. The relative migration area was measured using ImageJ software [17].

### 2.9. Transwell Assay

When GPM6A expression of cells was upregulated or downregulated, A549 (2.5 × 10^^4^) and BEAS-2B (2 × 10^^4^) cells in serum-free DMEM were inoculated evenly in the upper chambers of transwell plates, while DMEM containing 10% FBS was placed in the lower chambers for 24 h. The upper chambers were placed in 4% cold paraformaldehyde to fix the cells penetrating the membranes. The cells were then stained with 0.1% crystal violet. Photographs were taken using a light microscope, and the number of migrated cells was calculated using ImageJ.

### 2.10. Immunofluorescence Staining

After up-regulation or downregulation of GPM6A, the two cell lines were incubated with primary antibodies at 4°C overnight. Antibodies against anti-E-cadherin (1 : 50; rabbit polyclonal; AF0131; affinity), anti-N-cadherin (1 : 50; rabbit polyclonal; AF4039; affinity), and anti-vimentin (1 : 50; rabbit polyclonal; AF7013; affinity) were used. Secondary antibodies (goat anti-rabbit IgG; 1 : 1000; cat. no. ^#^A-21428; Thermo, USA) were used the next day. DAPI was used to stain the nuclei. Images of the stained cells were taken under a fluorescence microscope. Photographs were taken in six random fields at different magnifications.

### 2.11. Nude Mice Xenograft Tumor Assays

Ten male nude mice aged 5–6 weeks were obtained from Shanghai Jie Si Jie Laboratory Animal Co. Ltd. (Shanghai, China). The mice were randomly divided into the control group and the GPM6A overexpression group. Mice were subcutaneously injected with 1 × 10^^6^ cells (empty vector) or the same number of cells (Lv-GPM6A). The tumor size was first recorded on day 14 and then every 3 days thereafter. The mice were subsequently sacrificed on day 36, and the subcutaneous tumor was resected and weighed. Then, the tumor tissues were stored at −80°C.

This study was approved by the Ethics Review Board of Shanghai East Hospital at Tongji University (Shanghai, China). All animal experiments were performed in accordance with the Guide for the Care and Use of Laboratory Animals, published by the US National Institutes of Health.

### 2.12. Western Blot Analysis

A mixed solution of SDS lysis buffer, phenylmethylsulfonyl fluoride, protease, and phosphatase inhibitor was added in the two cell lines for complete proteolysis, and the superior solution was collected after centrifugation. After the protein concentrations were measured, the collected supernatants were separated by 10% sodium dodecyl sulfate polyacrylamide gel electrophoresis and transferred to a polyvinylidene difluoride membrane. After incubation with primary antibodies at 4°C overnight, the membrane was incubated with secondary antibodies (1 : 10000; goat anti-rabbit IgG; ab6721; Abcam) and exposed using an ECL assay kit [17].

The primary antibodies used were as follows: anti-GPM6A (1 : 1000; rabbit polyclonal; DF9699; Affinity Biosciences, Melbourne, Australia), anti-E-cadherin (1 : 1000; rabbit polyclonal; AF0131; Affinity), anti-N-cadherin (1 : 1000; rabbit polyclonal; AF4039; Affinity), anti-Zeb1(1 : 1000; rabbit polyclonal; AF7414; Affinity), anti-snail (1 : 1000; rabbit monoclonal; #3879; Cell Signaling Technology, Boston, USA), anti-vimentin (1 : 1000; rabbit polyclonal; AF7013; Affinity), anti-AKT (1 : 1000; rabbit monoclonal; #4691; Cell Signaling Technology), anti-phosphorylated AKT (p-AKT) (1 : 1000; rabbit polyclonal; ARG51558; Arigo biolaboratories, Shanghai, China), anti-phosphoinositide3-kinase (PI3K) (1 : 1000; rabbit monoclonal; ab191606; Abcam, Cambridge, UK), anti-phosphorylated-PI3K (p-PI3K) (1 : 1000, rabbit polyclonal; ab182651; Abcam), anti-ERK1/2(1 : 1000; rabbit monoclonal; #4695; Cell Signaling Technology), anti-phosphorylated-ERK1/2 (p-ERK1/2) (1 : 1000; rabbit monoclonal; #4370; Cell Signaling Technology), anti-STAT3(1 : 1000, rabbit monoclonal; ab68153; Abcam), anti-phosphorylated-STAT3 (p-STAT3) (1 : 1000, rabbit polyclonal; ab131103; Abcam), anti-*β*-catenin (1 : 1000; rabbit monoclonal; #8480; Cell Signaling Technology), anti-phosphorylated-*β*-catenin (p-*β*-catenin) (1 : 1000; rabbit monoclonal; #4176; Cell Signaling Technology), and anti-*β*-actin (1 : 1000; rabbit monoclonal; ^#^4970; Cell Signaling Technology).

### 2.13. Statistical Analysis

Data were presented as the mean ± standard deviation (SD). Each experiment was conducted three times independently. Data were compared between two groups using Student's *t*-test and among three groups using one-way analysis of variance. All statistical analyses were performed using GraphPad Prism 6.0 software. A *P* value of <0.05 was considered statistically significant.

## 3. Results

### 3.1. GPM6A Is Downregulated in Lung Adenocarcinoma

**Figure 1:**
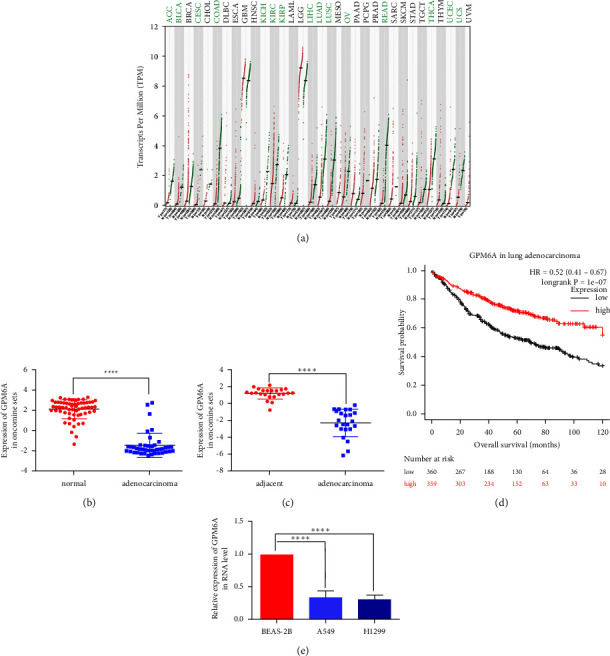
Comparison of GPM6A mRNA expression between tumors and corresponding noncancerous tissues. (a) GPM6A mRNA expression in tumors and noncancerous samples in 33 types of cancers in TCGA dataset (red lines: tumor samples; green lines: noncancerous samples). (b) mRNA GPM6A expression in lung adenocarcinoma and normal lung tissues in Hou Lung datasets from the Oncomine database. (c) mRNA GPM6A expression in lung adenocarcinoma and adjacent lung tissues in Su Lung datasets from the Oncomine database. (d) Kaplan–Meier survival analysis for lung adenocarcinoma patients with high and with low GPM6A expression (http://kmplot.com/analysis/) (ID:209469). (e) mRNA GPM6A expression in human lung adenocarcinoma cell lines (A549, H1299) and normal pulmonary epithelial cells (BEAS-2B). Data shown are the mean ± standard deviation (SD) for three independent experiments. (^*∗∗∗∗*^*P* < 0.0001).

GPM6A mRNA expression was lower in 15 types of cancers including lung adenocarcinoma (Figure 1(a)) in TCGA dataset. In the Oncomine dataset, GPM6A expression was significantly lower in lung adenocarcinoma tissues (42 samples) than in normal lung tissues (63 samples) (Hou lung) (Figure 1(b)). Similar results were obtained for comparison between 23 pairs of lung adenocarcinomas and adjacent lung tissues (Su lung) (Figure 1(c)). Furthermore, the Kaplan–Meier plot revealed that lung adenocarcinoma patients with lower GPM6A expression had worse prognosis (Figure 1(d)). RT-qPCR results showed lower GPM6A expression in A549 and H1299 than in BEAS-2B (Figure 1(e)).

### 3.2. GPM6A Inhibition Promotes Lung Adenocarcinoma Progression *In Vitro*

**Figure 2:**
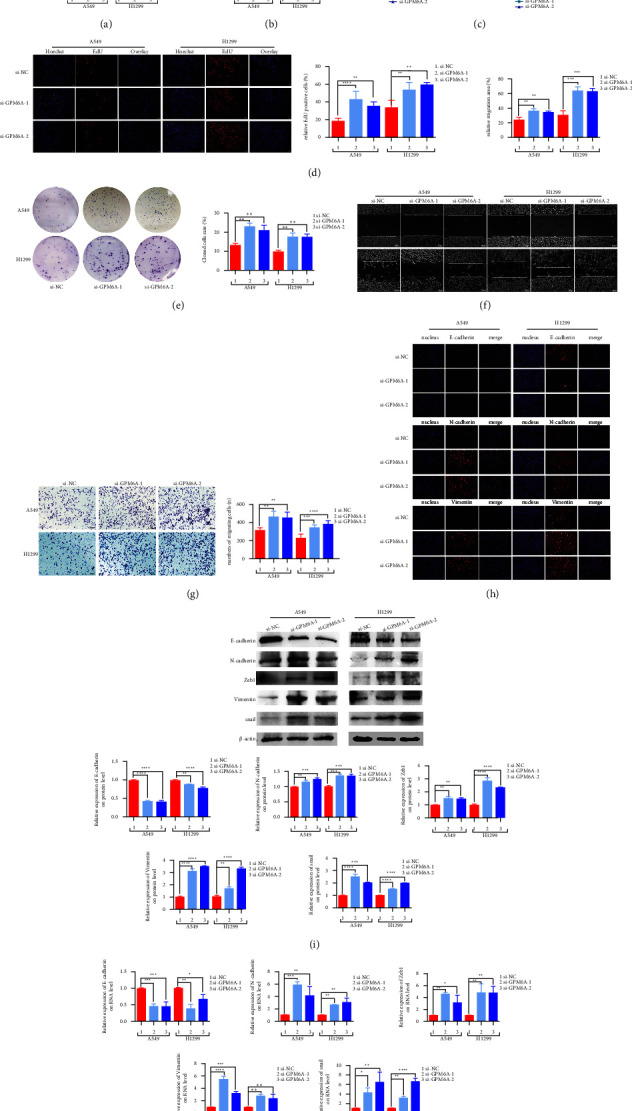
GPM6A suppression promotes the growth and migration of lung adenocarcinoma cells. (a) GPM6A mRNA expression in A549 and H1299 cells transfected with si-NC and si-GPM6A-1 and -2. (b) GPM6A protein expression in A549 and H1299 cells transfected with si-NC and si-GPM6A-1 and -2. (c) OD value (450 nm) of cells at 0, 24, 48, and 72 h of transfection with si-NC and si-GPM6A-1 and -2. (d) Relative number of EdU-positive cells after transfection with si-NC and si-GPM6A-1 and -2. (e) Colony-forming numbers of cells after transfection with si-NC and si-GPM6A-1 and -2. (f) Migration area of cells after transfection with si-NC and si-GPM6A-1 and -2. (g) Number of migrating cells after transfection with si-NC and si-GPM6A-1 and -2. (h) Immunofluorescence expression of EMT markers (E-cadherin, N-cadherin, and vimentin) after transfection with si-NC and si-GPM6A-1 and -2. (i) The expression of EMT markers (E-cadherin, N-cadherin, Zeb1, vimentin, and snail) on WB after transfection with si-NC and si-GPM6A-1 and -2. (j) Expression of EMT markers (E-cadherin, N-cadherin, Zeb1, vimentin, and snail) on RT-qPCR after transfection with si-NC and si-GPM6A-1 and -2. Data shown are the mean ± standard deviation (SD) for three independent experiments (^*∗*^*P* < 0.05, ^*∗∗*^*P* < 0.01, ^*∗∗∗*^*P* < 0.001, and ^*∗∗∗∗*^*P* < 0.0001) (OD: optical density and EMT: epithelial-mesenchymal transition).

RT-qPCR and WB results showed that GPM6A expression was lower in si-GPM6A-1,2-transfected cells than in si-NC treated cells (Figures 2(a) and 2(b)). The CCK8 assay showed that GPM6A downregulation promoted the growth of the two lung adenocarcinoma cell lines (Figure 2(c)). Similar results were observed in the EdU and colony formation assays (Figures 2(d) and 2(e)). The migration assays revealed that GPM6A downregulation significantly promoted the migration of A549 and H1299 cells (Figures 2(f) and 2(g)). Immunofluorescence assays revealed that GPM6A inhibition downregulated E-cadherin expression and upregulated N-cadherin and vimentin expression (Figure 2(h)). WB and RT-qPCR showed that GPM6A inhibition downregulated E-cadherin expression and upregulated N-cadherin, Zeb1, vimentin, and snail expression (Figures 2(i) and 2(j)). However, when GPM6A was inhibited (S1(a)), it had no effect on the proliferation, migration, and EMT of normal pulmonary epithelial cells (BEAS-2B) (S1(b)–S1(d)).

### 3.3. GPM6A Upregulation Inhibits Lung Adenocarcinoma Progression *In Vitro* and *In Vivo*

**Figure 3:**
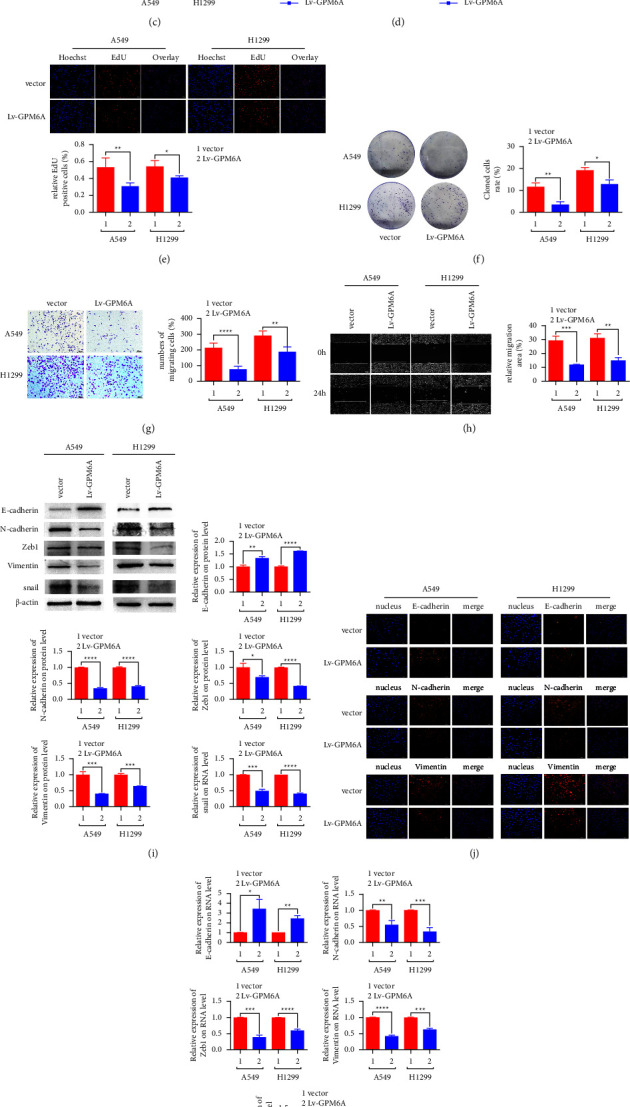
GPM6A upregulation inhibits the progression of lung adenocarcinoma. (a) GFP expression of A549 and H1299 under transfection with lentivirus packaging with overexpression plasmid (HBLV-h-GPM6A-3xflag-ZsGreen-PURO) (Lv-GPM6A) or control plasmid (HBLV-ZsGreen-PURO) (vector). (b) GPM6A mRNA expression upon transfection with Lv-GPM6A or empty vector. (c) GPM6A protein expression upon transfection with Lv-GPM6A or empty vector. (d) OD value (450 nm) of cells at 0, 24, 48, and 72 h after transfection with Lv-GPM6A or empty vector. (e) Relative number of EdU-positive cells after transfection with Lv-GPM6A or empty vector. (f) Number of colony-forming cells after transfection with Lv-GPM6A or empty vector. (g) Number of migrating cells after transfection with Lv-GPM6A or empty vector. (h) Migration area of cells after transfection with Lv-GPM6A or empty vector. (i) Expression of the EMT markers (E-cadherin, N-cadherin, Zeb1, vimentin, and snail) on WB after transfection with Lv-GPM6A or empty vector. (j) Immunofluorescence expression of EMT markers (E-cadherin, N-cadherin, and vimentin) after transfection with Lv-GPM6A or empty vector. (k) Expression of EMT markers (E-cadherin, N-cadherin, Zeb1, vimentin, and snail) on RT-qPCR after transfection with Lv-GPM6A or empty vector. (l) Tumor volume at different time points in nude mice injected with Lv-GPM6A or vector A549. (m) Tumor weight in nude mice injected with Lv-GPM6A or vector A549. Data shown are the mean ± standard deviation (SD) for three independent experiments. (^*∗*^*P* < 0.05, ^*∗∗*^*P* < 0.01, ^*∗∗∗*^*P* < 0.001, and ^*∗∗∗∗*^*P* < 0.0001) (GFP: green fluorescence protein).

The two lung adenocarcinoma cells were transfected with lentivirus packaging with overexpression plasmid (HBLV-h-GPM6A-3xflag-ZsGreen-PURO) (Lv-GPM6A) or control plasmid (HBLV-ZsGreen-PURO) (vector) for gain of function study. The transfected cells showed green fluorescence protein expression (Figure 3(a)). RT-qPCR and WB showed that GPM6A expression was obviously higher in cells transfected with Lv-GPM6A than in cells transfected with vector (Figure 3(b) and 3(c)). CCK-8, EdU, and colony formation assays showed that GPM6A upregulation inhibited the proliferation of lung adenocarcinoma cells (Figures 3(d)–3(f)). The transwell and wound healing assays showed that GPM6A upregulation suppressed the migration abilities of lung adenocarcinoma cells (Figures 3(g) and 3(h)). WB and RT-qPCR indicated that GPM6A overexpression increased E-cadherin expression and decreased N-cadherin, Zeb1, vimentin, and snail expressions (Figures 3(i)–3(k)). Immunofluorescence assays revealed that upregulation of GPM6A increased E-cadherin expression and decreased N-cadherin and vimentin expressions (Figure 3(j)). However, when GPM6A was upregulated (S2(a)–S2(b)), it still had no effect on the proliferation, migration, and EMT of BEAS-2B (S2(c)–S2(e)). Furthermore, GPM6A upregulation delayed tumor growth in nude mice subcutaneously injected with Lv-GPM6A or vector-transfected A549 (Figure 3(l)). The volume and weight of tumors were also decreased in nude mice injected with A549 cells overexpressing GPM6A (Figure 3(m)). These results support that GPM6A overexpression suppresses the proliferation and metastasis of lung adenocarcinoma.

### 3.4. GPM6A Suppresses Lung Adenocarcinoma Progression via Inhibition of the PI3K/AKT Pathway

**Figure 4:**
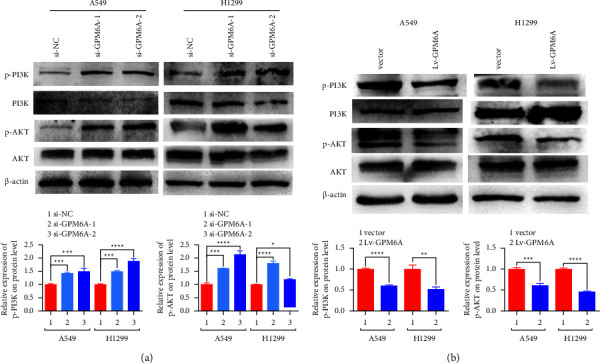
GPM6A suppresses lung adenocarcinoma development via inhibition of the PI3K/AKT pathway. (a) Expressions of PI3K, p-PI3K, AKT, and p-AKT at the protein level in cells transfected with si-NC and si-GPM6A-1 and -2. (b) Expressions of PI3K, p-PI3K, AKT, and p-AKT at the protein level in cells transfected with Lv-GPM6A and empty vector. Data shown are the mean ± standard deviation (SD) for three independent experiments (^*∗*^*P* < 0.05, ^*∗∗*^*P* < 0.01, ^*∗∗∗*^*P* < 0.001, and ^*∗∗∗∗*^*P* < 0.0001) (p-PI3K: phosphorylated PI3K and p-AKT: phosphorylated-AKT).

We further investigated the possible mechanism of GPM6A in lung adenocarcinoma cells. We chose the typical tumor signal pathways including the PI3K/AKT signaling pathway, MAPK signaling pathway, JAK/STAT signaling pathway, and Wnt/*β*-cateninsignaling pathway to find out the possible pathway. We detected PI3K, p-PI3K, AKT, p-AKT, STAT3, p-STAT3, ERK1/2, p-ERK1/2, *β*-catenin, and p-*β*-catenin expressions by WB. The results showed that PI3K and AKT were activated upon GPM6A inhibition (Figure 4(a)). In contrast, PI3K and AKT were inhibited upon GPM6A upregulation (Figure 4(b)). However, we did not find the differential expression of STAT3, p-STAT3, ERK1/2, p-ERK1/2, *β*-catenin, and p-*β*-catenin in A549 transfected with Lv-GPM6A and vector (S3). These findings show that GPM6A might suppress lung adenocarcinoma proliferation via inhibition of the PI3K/AKT pathway.

## 4. Discussion

The role of GPM6A in lung cancer is yet to be clarified. In the current study, GPM6A mRNA expression was lower in 15 types of tumors including lung adenocarcinoma in TCGA dataset. GPM6A expression was also lower in lung adenocarcinoma than in adjacent tissues in the Oncomine database. GPM6A downregulation enhanced the proliferation and migration of lung adenocarcinoma cells, while GPM6A upregulation suppressed the proliferation and metastasis of lung adenocarcinoma *in vitro* and *in vivo.* Furthermore, GPM6A knockdown activated the PI3K/AKT pathway, whereas GPM6A upregulation inactivated the PI3K/AKT pathway.

GPM6A is a stress-responsive gene belonging to the proteolipid protein family. It is expressed on neuronal membrane proteins in the CNS. It binds to the *μ*-opioid receptor and regulates immune response and stress [18]. Alvarez Juliá et al. reported that GPM6A is involved in neurite extension, filopodium and spine formation, and synaptogenesis under stress [19]. Michibata et al. also showed that GPM6A regulates the opiate drug receptor during treatment of chronic stress and pain [20]. Recent studies reported that GPM6A is associated with various diseases. In our study, TCGA dataset results showed that GPM6A mRNA expression was obviously decreased in 15 types of tumors and increased in 18 types of tumors. Therefore, its role in tumor is ambiguous.

Liu et al. found low GPM6A expression in HCC, and GPM6A downregulation regulated the cell cycle and promoted the development of HCC by activating the AKT/ERK pathway [14]. Cai et al. analysed TCGA dataset and found that rectal cancer patients with low GPM6A expression had longer overall survival time [21]. However, studies on the relevance of GPM6A in lung cancer are limited. Jiang et al. reported that GPM6A was inhibited in small‐cell lung cancer cells, which was regulated by miR-22 [15]. In the current study, GPM6A expression at the mRNA level was lower in lung adenocarcinoma than in normal or adjacent lung tissues. Further function study showed that GPM6A inhibits the propagation and migration of lung adenocarcinoma cells. These findings indicated that GPM6A acts as a tumor suppressor.

The PI3Ks are a large family of lipid enzymes that phosphorylate the 3′-OH group of phosphatidylinositols on the plasma membrane [22]. AKT is one of the major downstream effectors of PI3K. The signaling pathway participates in many aspects of cell growth and survival in physiological and pathological conditions [23]. The PI3K/AKT pathway is a key regulator of survival during cellular stress. As tumors exist in a stressful environment, this pathway plays a crucial role in the development of cancer [23]. Given that GPM6A is associated with stress, we detected PI3K, p-PI3K, AKT, and p-AKT expression at the protein level and found that GPM6A downregulation promoted the progression of lung adenocarcinoma cells. In contrast, GPM6A upregulation inhibited the progression of lung cancer. Although we detected the other typical tumor signaling pathways including MAPK, JAK/STAT3, and Wnt/*β*-catenin signaling pathways, it had no differential expression between Lv-GPM6A and the empty vector. Therefore, GPM6A might suppress lung adenocarcinoma progression via inhibition of the PI3K/AKT pathway.

This study has some limitations. We did not investigate the function of GPM6A in PI3K inhibitor treatment. The role of GPM6A inhibition in lung adenocarcinoma was also not observed *in vivo.* These will be addressed in our future research.

## 5. Conclusions

In conclusion, GPM6A acts as a tumor suppressor and inhibits the proliferation and migration of lung adenocarcinoma via inhibition of the PI3K/AKT pathway. Therefore, GPM6A could be a possible treatment target for lung cancer therapy.

## Figures and Tables

**Table 1:** Primer sequences.

GPM6A	5′-TCTGCCGGAACACCACATTAG-3′	5′-GTAGATTCGCACATCCTCAAGAA-3′
E-cadherin	5′-CGAGAGCTACACGTTCACGG-3′	5′-GGGTGTCGAGGGAAAAATAGG-3′
N-cadherin	5′-TGCGGTACAGTGTAACTGGG-3′	5′-GAAACCGGGCTATCTGCTCG-3′
Zeb1	5′-GATGATGAATGCGAGTCAGATGC-3′	5′-ACAGCAGTGTCTTGTTGTTGT -3′
Vimentin	5′-GACGCCATCAACACCGAGTT-3′	5′-CTTTGTCGTTGGTTAGCTGGT-3′
Snail	5′-TCGGAAGCCTAACTACAGCGA -3′	5′-AGATGAGCATTGGCAGCGAG -3′
GAPDH	5′-GAGTCAACGGATTTGGTCGT-3′	5′-TTGATTTTGGAGGGATCTCG-3′
si-NC	5′-UUCUCCGAACGUGUCACGUTT-3′	5′-ACGUGACACGUUCGGAGAATT-3′
si-GPM6A-1	5′-CCAUGAUUGACAUCUUUAATT-3′	5′-UUAAAGAUGUCAAUCAUGGTT-3′
si-GPM6A-2	5′-GGCCAUCAAAGAUCUCUAUTT-3′	5′-AUAGAGAUCUUUGAUGGCCTT-3′

## Data Availability

The data used to support the findings of this study are available from the corresponding author upon request.

## Supplementary Materials

Supplementary Materials S1:GPM6A suppression has no effect on BEAS-2B. (A) GPM6A mRNA expression in BEAS-2B cell transfected with si-NC and si-GPM6A-1 and -2. (B) OD value (450 nm) of cells at 0, 24, 48, and 72 h of transfection with si-NC and si-GPM6A-1 and -2. (C) Migration area of cells after transfection with si-NC and si-GPM6A-1 and -2. (D) Expression of EMT markers (E-cadherin, N-cadherin, Zeb1, vimentin, and snail) on RT-qPCR after transfection with si-NC and si-GPM6A-1 and -2. Data shown are the mean ± standard deviation (SD) for three independent experiments (^*∗∗∗∗*^*P* < 0.0001). S2: GPM6A upregulation has no effect on BEAS-2B. (A) GFP expression of BEAS-2B under transfection with lentivirus packaging with overexpression plasmid (HBLV-h-GPM6A-3xflag-ZsGreen-PURO) (Lv-GPM6A) or control plasmid (HBLV-ZsGreen-PURO) (vector). (B) GPM6A mRNA expression upon transfection with Lv-GPM6A and empty vector. (C) OD value (450 nm) of cells at 0, 24, 48, and 72 h of transfection with Lv-GPM6A and empty vector. (D) Migration area of cells after transfection with Lv-GPM6A and empty vector. (E) Expression of EMT markers (E-cadherin, N-cadherin, Zeb1, vimentin, and snail) on RT-qPCR after transfection with Lv-GPM6A and empty vector. Data shown are the mean ± standard deviation (SD) for three independent experiments. S3: expressions of STAT3, p-STAT3, ERK1/2, p-ERK1/2, *β*-catenin, and p-*β*-catenin at the protein level in A549 transfected with Lv-GPM6A and empty vector. Data shown are the mean ± standard deviation (SD) for three independent experiments.
